# Outpatient physical therapy bundled payment models are feasible for total hip arthroplasty patients: an evaluation of utilization, cost and outcomes

**DOI:** 10.1186/s42836-023-00179-2

**Published:** 2023-05-12

**Authors:** Laura A. Stock, Andrea H. Johnson, Jane C. Brennan, Justin J. Turcotte, Paul J. King, James H. MacDonald

**Affiliations:** Anne Arundel Medical Center, Annapolis, MD 21401 USA

**Keywords:** Total hip arthroplasty (THA), Physical therapy (PT), Bundled payment

## Abstract

**Background:**

Various episode-of-care bundled payment models for patients undergoing total joint arthroplasty have been implemented. However, participation in bundled payment programs has dropped given the challenges of meeting continually lower target prices. The purpose of our study is to investigate the cost of outpatient physical therapy (PT) and the potential for stand-alone outpatient PT bundled payments for patients undergoing total hip arthroplasty (THA).

**Methods:**

A retrospective review of 501 patients who underwent primary unilateral THA from November 2017 to February 2020 was performed. All patients included in this study received postoperative PT care at a single hospital-affiliated PT practice. Patients above the 75th percentile of therapy visits were then classified as high-PT utilizers and compared with the rest of the population using univariate statistics. Stepwise multivariate logistic regression was used to assess the predictors of high therapy utilization.

**Results:**

Patients averaged 65 ± 10 years of age and a BMI of 29 ± 5 kg/m^2^. Overall, 80% of patients were white and 53% were female. The average patient had 11 ± 8 total therapy sessions in 42 days: one initial evaluation, one re-evaluation and 9 standard sessions. High-PT utilizers incurred estimated average costs of $1934 ± 431 per patient, compared to $783 ± 432 (*P* < 0.001) in the rest of the population. Further, no significant differences in 90-day outcomes including lower extremity functional scale scores, emergency department returns, readmissions, or returns to the operating room were observed between high utilizers and the rest of the population (all *P* > 0.08). In the multivariate analysis, women (OR = 1.68, *P* = 0.017) and those with sleep apnea (OR = 2.02, *P* = 0.012) were nearly twice as likely to be high utilizers, while white patients were 42% less likely to be high utilizers than patients of other races (OR = 0.58, *P* = 0.028).

**Conclusions:**

Outpatient PT utilization is highly variable in patients undergoing THA. However, despite using more services and incurring increased cost, patients in the top quartile of utilization experienced similar outcomes to the rest of the population. If outpatient therapy bundles are to be developed, 16 visits appear to be a reasonable target for pricing, given this provides adequate coverage for 75% of THA patients.

**Supplementary Information:**

The online version contains supplementary material available at 10.1186/s42836-023-00179-2.

## Background

Physical therapy (PT) is an important step in any treatment protocol following total joint arthroplasty (TJA) to improve a patient’s mobility, strength, and independence. In patients undergoing total hip arthroplasty (THA), PT is typically prescribed for 2 to 3 days a week for 6 to 8 weeks [1]. Therapy often starts the day of or the day after surgery and continues for two to three days a week along with home exercises until activity goals are met [2]. As the performance of TJA continues to shift toward the ambulatory setting, more intensive early therapy programs are being implemented to facilitate early mobilization and same-day discharge [3–6]. Such therapy protocols have been shown to decrease hospital length of stay (LOS), decrease hospital costs, increase patient satisfaction, and improve functional status more rapidly [3–6]. Additionally, the costs and benefits of various PT models, including formal outpatient, home-based, and no therapy, have been evaluated [7–10]. With some recent studies showing that formal outpatient PT was not necessary for all THA patients [10, 11], additional research into new models that maximize the value of traditional therapy is needed in order to maintain access to these services.

Outpatient PT represents a significant portion of TJA episode cost [11]. In an effort to reduce cost, bundled payment initiatives such as the Comprehensive Care for Joint Replacement (CJR), Bundled Payments for Care Improvement (BPCI), and BPCI Advanced programs have been put in place [12–15]. While the mechanics of each program differ, they operate in a similar manner by incentivizing hospitals and/or providers to deliver care under an established target price while meeting quality thresholds. Upon implementation, these programs effectively reduced the costs of TJA, primarily through reductions in LOS and discharge to skilled nursing facilities (SNFs) [13, 16, 17]. Despite these promising early results, participation in bundled payment programs has dropped given the challenges of meeting continually lower target prices [12, 18]. With target pricing decreasing by the CMS, the withdrawal rate of the BPCI Advanced programs has risen to over 85% [19]. As the financial savings and quality improvement gains from participation in episode of care bundled payment programs slow, the ultimate fate of such programs remains uncertain [19, 20].

In light of the debate over the value of formal outpatient PT and the challenges of successfully managing the cost of an entire episode of care, alternative value-based payment models, such as bundled pricing for a distinct phase of care may be more attractive to providers. The purpose of our study was to investigate the cost of outpatient PT and the potential effectiveness of stand-alone outpatient PT payment bundles for patients undergoing THA.

## Methods

This study was deemed institutional review board exempt as a review of existing medical records by the institutional clinical research committee, and a waiver of informed consent was granted. A retrospective chart review of all patients undergoing THA by 7 board certified surgeons at a single institution was performed.

### Study population

All patients included in this study underwent primary unilateral THA from November 2017 to February 2020. Patients undergoing bilateral or revision THA were excluded from this study. All patients included in this study received postoperative PT care at a single hospital-affiliated practice. A total of 501 patients met the inclusion criteria.

### Perioperative protocol

All patients were cared for in a coordinated Joint Replacement Center and received education materials including written materials, preoperative medical evaluations, preoperative home exercise or outpatient physical therapy, and an education class for patients and their caregivers. All patients were treated utilizing a multimodal pain management protocol which, depending on patient factors, included acetaminophen, oral NSAIDs, pregabalin, ketorolac, and oral opioid medications as needed.

### PT protocol

Standard PT protocols are used across all therapy sites. However, therapists might modify treatment based on their clinical judgment of patient progression. During weeks 0–2, therapy focuses on range of motion (ROM), flexibility, quadriceps strengthening exercises and gait training. During this period, patients were expected to transition from walker to cane-assisted ambulation. During weeks 3–6, scar mobilization is initiated and assistive devices were discontinued as the patient’s gait normalizes. In this phase, exercises focus on quadriceps, hamstring and core strengthening, hip abduction and adduction, and proprioception. During weeks 7–12, therapy focused on continued strengthening, single leg stance and uneven terrain exercises, and gait training with the goal of mastering functional activities, improving strength, and normalizing gait patterns. Finally, in weeks 13–16 intense lower extremity weight training and sport-specific training programs began with the goals of approximating muscle strength and returning to sport-specific activities.

### Independent variables

Data were collected using an administrative database for patient demographics, including age, sex, race, and body mass index (BMI). Seventeen comorbidities (presented in Table 1) were evaluated as defined by International Classification of Disease 10th Edition (ICD-10) diagnosis codes. The definitions of each comorbidity used are presented in the Additional file 1. American Society of Anesthesiologists (ASA) score was used to quantify preoperative health status. The Centers for Medicare and Medicaid Services (CMS) Hierarchical condition category (HCC) score was also used to quantify levels of comorbidity burden. HCC quantifies patient health status by assigning risk scores to patients based on diagnosis codes and demographic factors and was calculated for risk stratification of all patients in a payer-agnostic fashion at our institution [21]. The HCC model sums demographic factors and disease-based condition categories based on diagnoses in the past year and applies an interaction factor to adjust for increased risk in patients with multiple related comorbidities. Scores are normalized to 1.0, with higher scores indicating a greater comorbidity burden and higher expected medical expenditures [22, 23]. The primary reason for THA was evaluated via manual chart review and classified as osteoarthritis (OA), fracture, avascular necrosis (AVN), or others.

**Table 1:** Total population demographics and comorbidities

Patient demographics	All patients (*n* = 501)
**Age**	65.37 ± 10.06
**Sex**	
Female	267 (53.3)
Male	234 (46.7)
**White race**	399 (79.6)
**BMI**	29.29 ± 5.39
**ASA 3 + **	167 (33.3)
**OA**	482 (96.2)
**Fracture**	10 (2.0)
**AVN**	9 (1.8)
**Other diagnoses**	0 (0)
**Obesity**	210 (41.9)
**Diabetes**	
Type 1 diabetes	1 (0.2)
Type 2 diabetes	61 (12.2)
Type 1 or 2 diabetes	62 (12.4)
**Sleep apnea**	72 (14.4)
**COPD**	20 (4.0)
**Liver disease**	10 (2.0)
**Asthma**	46 (9.2)
**AFIB**	30 (6.0)
**CHF**	3 (0.6)
**CAD**	48 (9.6)
**ESRD CKD**	29 (5.8)
**GERD**	144 (28.7)
**Anxiety/Depression**	106 (21.2)
**HTN**	260 (51.9)
**PVD**	0 (0)
**Neoplasm**	11 (2.2)
**Anemia**	12 (2.4)
**HCC score**	0.49 ± 0.27

*ASA* American Society of Anesthesiologisits, *AVN* Avascular necrosis, *OA* Osteoarthritis, *COPD* Chronic obstructive pulmonary disease, *AFIB* Atrial fibrillation, *CHF* Congestive heart failure, *CAD* Coronary artery disease, *ESRD CKD* End-stage renal disease/chronic kidney disease, *GERD* Gastroesophageal reflux disease, *HTN* Hypertension, *PVD* Peripheral vascular disease, *HCC* Hierarchical condition category

### Outcome measures

Outcomes of interest included the total number of therapy sessions, more than 3 months of PT, number of evaluations in 3 months, number of re-evaluations in 3 months, the total number of sessions, total therapy charge, days in PT, last lower extremity functional score (LEFS) within 3 months postoperatively, days to LEFS, 90-day emergency department return, 90-day readmission and 90-day return to the operating room (OR). All outcome measures were captured by manual review of the electronic medical record. Emergency department returns and readmissions included returns to outside institutions participating in the Epic Care Everywhere program. Physical therapy charges were estimated based on the CMS Medicare Multiple Procedure Payment Reduction (MPPR) 2022 Rate File [24]. Allowable amounts were calculated using the carrier and locality codes of our institution, and the 50% rate reduction was applied to all secondary and tertiary current procedure terminology (CPT) codes. CPT codes were selected based on the most commonly used treatment modalities for the various visit types at our institution. Using this approach, charges for the three types of therapy visits were estimated as follows: evaluation (CPTs 97,161 [low complexity evaluation] + 97,116 [gait training] + 97,140 [manual therapy], $181.01), re-evaluation (CPTs 97,164 [re-evaluation est. plan of care] + 97,110 [therapeutic exercises] + 97,116 + 97,140, $147.26), non-evaluation/re-evaluation treatment session (CPTs 97,110 + 97,116 + 97,140, $79.43).

### Statistical analysis

Descriptive statistics were used to determine the prevalence of demographics, comorbidities, outcomes, and utilization patterns of all patients. Patients were then classified based on the number of total PT sessions. The top quartile of high utilizers requiring over 16 PT sessions was compared to the rest of the population. The top quartile was selected as the threshold for 'high-utilizers' in alignment with prior studies, as the top 25% of healthcare utilizers account for a disproportionate amount of aggregate healthcare spend [25–27]. As depicted in Tables 4 and 5, univariate analyses, including chi-square tests and independent samples *t*-tests, were used to determine demographic, comorbidity, utilization, and outcome differences between these groups. The Fisher’s Exact test and Mann Whitney U test were performed when the assumptions of chi-square and independent samples *t*-testing were not met. Stepwise multivariate logistic regression was used to assess the predictors of having more than 16 total sessions. Subgroup analysis was then performed to compare outcomes across PT utilization quartiles. One-way ANOVA and chi-square testing were performed, with non-parametric tests used when the assumptions of parametric testing were not met. All statistical analyses were performed using R Studio (Version 1.4.1717© 2009–2021 RStudio, PBC). Statistical significance was assessed at *P* < 0.05.

## Results

Patients included in the study averaged 65 ± 10 years of age and a BMI of 29 ± 5 kg/m^2^. Overall, 80% of patients were white and 53% were female. The majority (96%) of patients underwent THA for the treatment of OA. The most common comorbidities observed were hypertension (HTN) in 52% of patients, obesity (42%), and gastroesophageal reflux disease (GERD, 29%). The prevalence of specific comorbidities is listed in Table 1.

**Fig. 1:**
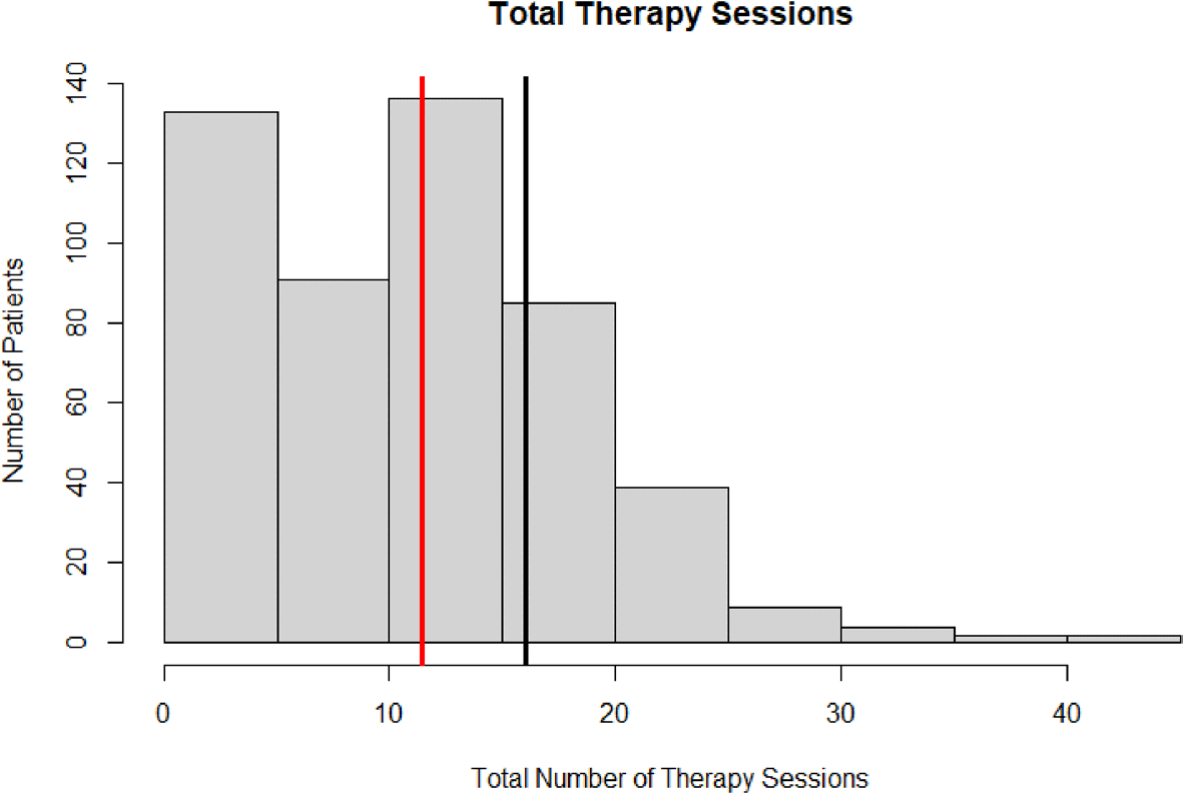
Histogram of total number of therapy sessions: The red line depicts the mean number of therapy session utilized by the population. The black line depicts the 75th percentile threshold of 16 sessions used to classify high-PT utilizers

**Fig. 2:**
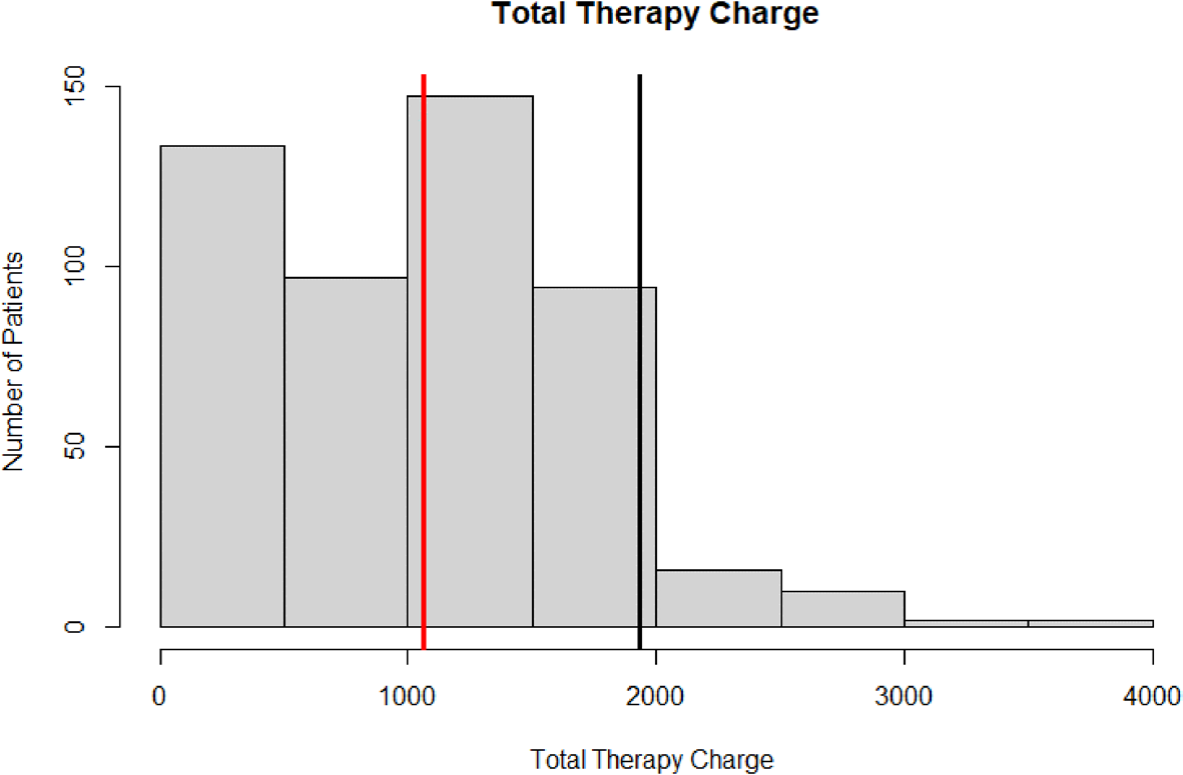
Total therapy charge: The red line depicts the mean therapy charges of the population. The black line depicts the 75th percentile of therapy charges incurred by high-PT utilizers

Across the entire population, patients utilized an average of 11.45 ± 7.69 PT visits over the three-month postoperative period, accounting for $1065.43 ± 657.48 in charges (Figs. 1 and 2). On average, these included one evaluation session, one re-evaluation session, and 9.50 standard therapy sessions. The average time in PT was 42.27 ± 31.95 days and 7.4% of patients required over 3 months of therapy. Over the 3-month postoperative period, the last average LEFS was 30.45 ± 18.79. Unplanned resource utilization, including 90-day ED returns, readmissions, and returns to the OR occurred in 5.0%, 6.8%, and 4.4% of patients, respectively (Table 2).

**Table 2:** PT utilization and outcomes

Outcomes	All patients (*n* = 501)
Total number of therapy sessions (0–3 months)	11.45 ± 7.69
3 + months in PT	37 (7.4)
# Evaluations (0–3 months)	1.00 ± 0
# Re-evaluations (0–3 months)	0.95 ± 0.96
Total number of PT sessions	9.50 ± 6.88
Total therapy charge ($)	1065.43 ± 657.48
Days in PT	42.27 ± 31.95
Last 90-day LEFS ^a^	30.45 ± 18.79
Days to LEFS	19.50 ± 23.69
90-day ED return	25 (5.0)
90-day readmission	34 (6.8)
90-day return to OR	22 (4.4)

Data are expressed as mean ± SD or* n* (%); ^a^: *n* = 203; *PT* Physical therapy, *LEFS* Lower extremity functional score, *ED* Emergency department, *OR* Operating room

In comparison to the rest of the population, high-PT utilizers requiring 16 or more sessions were significantly more likely to be female (61.8% vs. 50.5%, *P* = 0.038) and have sleep apnea (20.3% vs. 12.4%, *P* = 0.043). No statistically significant differences in demographics or comorbidities were observed between the high-PT utilizer group and the rest of the population (Table 3). As expected, high-PT utilizers required more overall sessions on average (21.6 ± 5.03 vs. 8.15 ± 5.05, *P* < 0.001) and incurred higher charges ($1933.94 ± 431.20 vs. 782.82 ± 431.70, *P* < 0.001) than the rest of the population. Further, a significantly higher proportion of high-utilizers required over 3 months of PT (28.5% vs. 0.5%). No statistically significant differences in last LEFS scores were observed in high-PT utilizers (35.0 ± 21.4 vs. 30.5 ± 20.6, *P* = 0.199), despite the fact that the time to last LEFS was significantly longer in this group than the rest of the population (34.7 ± 28.8 days vs. 14.3 ± 19.1 days). No statistically significant differences in rates of 90-day ED returns, readmissions, or returns to OR were observed between groups (Table 4).

**Table 3:** Patient demographics and comorbidities

Demographics and comorbidities	Less than or 16 Sessions (*n* = 378)	16 + Sessions (*n* = 123)	*P*-value
**Age**	65.07 ± 10.02	66.31 ± 10.15	0.239
**Sex**			**0.038**
Female	191 (50.5)	76 (61.8)	
Male	187 (49.5)	47 (38.2)	
**White race**	309 (81.7)	90 (73.2)	0.055
**BMI**	29.18 ± 5.31	29.66 ± 5.62	0.410
**ASA 3 + **	126 (33.3)	41 (33.3)	1
**OA**	366 (96.8)	116 (94.3)	0.274
**Fracture**	5 (1.3)	5 (4.1)	0.071*
**AVN**	7 (1.9)	2 (1.6)	1*
**Obesity**	153 (40.5)	57 (46.3)	0.298
**Diabetes**			
Type 1 diabetes	1 (0.3)	0 (0)	1*
Type 2 diabetes	46 (12.2)	15 (12.2)	1
Type 1 or 2 diabetes	47 (12.4)	15 (12.2)	1
**Sleep apnea**	47 (12.4)	25 (20.3)	**0.043**
**COPD**	14 (3.7)	6 (4.9)	0.597
**Liver disease**	7 (1.9)	3 (2.4)	0.713*
**Asthma**	33 (8.7)	13 (10.6)	0.665
**AFIB**	23 (6.1)	7 (5.7)	1
**CHF**	2 (0.6)	1 (0.8)	0.571*
**CAD**	38 (10.1)	10 (8.1)	0.651
**ESRD CKD**	23 (6.1)	6 (4.9)	0.783
**GERD**	109 (28.8)	35 (28.5)	1
**Anxiety/depression**	78 (20.6)	28 (22.8)	0.708
**HTN**	192 (50.8)	68 (55.3)	0.446
**PVD**	0 (0)	0 (0)	1
**Neoplasm**	7 (1.9)	4 (3.3)	0.476*
**Anemia**	8 (2.1)	4 (3.3)	0.500*
**HCC score**	0.49 ± 0.29	0.48 ± 0.21	1

*P*-values < 0.05 are in bold; * Denotes Fisher’s Exact Test; Data are expressed as mean ± SD or* n* (%); *BMI* Body mass index, *ASA* American Society of Anesthesiologists, *AVN* Avascular necrosis, *OA* Osteoarthritis, *COPD* Chronic obstructive pulmonary disease, *AFIB* Atrial fibrillation, *CHF* Congestive heart failure, *CAD* Coronary artery disease, *ESRD CKD* End-stage renal disease/chronic kidney disease, *GERD* Gastroesophageal reflux disease, *HTN* Hypertension, *PVD* Peripheral vascular disease, *HCC* Hierarchical condition category

**Table 4:** Outcomes by PT utilization

Outcomes	Less than or 16 sessions (*n* = 378)	16 + Total sessions (*n* = 123)	*P*-value
Total number of sessions (0–3 months)	8.15 ± 5.05	21.6 ± 5.03	** < 0.001**
3 + months in PT	2 (0.5)	35 (28.5)	** < 0.001**^a^
# Evaluations (0–3 months)	1.00 ± 0	1.00 ± 0	1
# Re-evaluations (0–3 months)	0.58 ± 0.62	2.08 ± 0.94	** < 0.001**
Total # of PT sessions	6.57 ± 4.59	18.5 ± 4.49	** < 0.001**
Total PT charge ($)	782.82 ± 431.7	1933.94 ± 431.2	** < 0.001**
Evaluation PT charges	181.01 ± 0	181.01 ± 0	1
Re-evaluation PT Charges	80.3 ± 84.8	281.01 ± 114.5	** < 0.001**
Days in PT	29.6 ± 22.1	81.1 ± 25.9	** < 0.001**
Last 90-day LEFS	30.5 ± 20.6 ^b^	35.0 ± 21.4 ^c^	0.199
Days to LEFS	14.3 ± 19.1	34.7 ± 28.8	** < 0.001**
90-day ED return	23 (6.1)	2 (1.6)	0.083^a^
90-day readmission	29 (7.7)	5 (4.1)	0.240
90-day return to OR	19 (5.0)	3 (2.4)	0.179

*P*-value < 0.05 are in bold; Data are expressed as mean ± SD or* n* (%); ^a^Denotes Fisher’s Exact Test; ^b^ *n* = 151, ^c^ *n* = 52; *PT* Physical therapy, *LEFS* Lower extremity functional score, *ED* Emergency department, *OR* Operating room

In the subgroup analysis of the population stratified in quartiles of therapy utilization, statistically significant differences in all cost and utilization measures were observed, with the exception of the number of re-evaluations and re-evaluation charges (all *P* < 0.001). As expected, each increasing quartile of therapy utilization incurred higher charges and resulted in longer therapy duration. Across the utilization quartiles, significant differences in the last LEFS scores (*P* = 0.025) and time to last LEFS score (*P* < 0.001) existed. Notably, the highest levels of postoperative function were observed in the third quartile of patients undergoing 11–15 total PT sessions. Finally, no statistically significant differences in rates of 90-day ED returns, readmissions, or returns to OR were observed across therapy utilization quartiles (Table 5).

**Table 5:** Outcomes by PT utilization quartiles

**Outcomes**	**Quartile 1:** ≤ 25th percentile (< 5 total sessions, *n* = 122)	**Quartile 2:** 26-50th percentile (5–10 total sessions, *n* = 122)	**Quartile 3:** 51-74th percentile (11–15 total sessions, *n* = 134)	**Quartile 4:** ≥ 75th percentile (16 + total sessions, *n* = 123)	***P*-value**
Total number of sessions (0–3 months)	1.89 ± 0.95	8.46 ± 1.97	13.6 ± 1.55	21.62 ± 5.03	** < 0.001**^b^
3 + months in PT	0 (0)	0 (0)	2 (1.5)	35 (28.5)	** < 0.001**^a^
# Evaluations (0–3 months)	1.00 ± 0	1.00 ± 0	1.00 ± 0	1.00 ± 0	1^b^
# Re-evaluations 0–3 months	0.02 ± 0.13	0.52 ± 0.53	1.14 ± 0.45	2.08 ± 0.94	** < 0.001**^b^
Total number of PT sessions	0.87 ± 0.93	6.93 ± 1.73	11.4 ± 1.58	18. 54 ± 4.49	** < 0.001**^b^
Total PT charge ($)	251.88 ± 76.73	803.62 ± 178.24	1248.59 ± 431.21	1933.95 ± 430.85	** < 0.001**^b^
Evaluation PT charges	181.01 ± 0	181.01 ± 0	181.01 ± 0	181.01 ± 0	1^b^
Re-evaluation PT charges	1.86 ± 15.1	71.0 ± 72.6	160.31 ± 57.2	280.57 ± 114.1	** < 0.001**^b^
Days in PT	4.20 ± 6.93	31.5 ± 11.1	51.1 ± 12.7	81.07 ± 25.89	** < 0.001**^b^
Last 90-day LEFS	22.9 ± 11.2 ^c^	31.5 ± 23.5 ^d^	36.5 ± 22.4 ^e^	35.0 ± 21.4 ^f^	**0.025**^b^
Days to LEFS	7.56 ± 15.6	14.8 ± 18.8	19.9 ± 20.7	34.7 ± 28.8	** < 0.001**^b^
90-day ED return	7 (5.7)	9 (7.4)	7 (5.2)	2 (1.6)	0.165^a^
90-day readmission	10 (8.2)	8 (6.6)	11 (8.2)	5 (4.1)	0.519
90-day return to OR	5 (4.1)	6 (4.9)	8 (5.9)	3 (2.4)	0.548

*P*-value < 0.05 are in bold; Data are expressed as mean ± SD or* n* (%); ^a^Denotes Fisher’s Exact Test; ^b^ Denotes Kruskal Wallis Test; ^c^ *n* = 48, ^d^ *n* = 51, ^e^ *n* = 52, ^f^* n* = 52; *PT* Physical therapy, *LEFS* Lower extremity functional score, *ED* Emergency department, *OR* Operating room

In the multivariate analysis, women (OR = 1.68, *P* = 0.017) and those with sleep apnea (OR = 2.02, *P* = 0.012) were nearly twice as likely to be high-PT utilizers requiring 16 or more sessions. However, the white race was protective against high therapy utilization (OR = 0.58, *P* = 0.028); white patients were 42% less likely to have over 16 sessions than non-white patients (Table 6).

**Table 6:** Stepwise multivariate logistic regression: predictors of 16 + sessions

Predictors	Odd ratio	95% Confidence interval	*P*-value
Female	1.68	1.10–2.60	**0.017**
White race	0.58	0.36–0.95	**0.028**
Fracture	3.17	0.86–11.65	0.074
Sleep apnea	2.02	1.15–3.47	**0.012**

*P*-value < 0.05 are in bold

## Discussion

The results of this study demonstrated that utilization of PT services after primary THA was highly variable, with the top quartile of high-PT utilizers averaging nearly 22 sessions postoperatively. These high utilizers were more likely to be female and have sleep apnea, and less likely to be of white race. While high levels of PT utilization were associated with increased costs, they did not translate to significantly improved physical function or decreased ED returns, readmissions, or returns to the OR during the 90-day postoperative period. With 75% of patients requiring 16 or fewer PT sessions, we suggest this threshold may be considered as a target for the development of future PT bundled payment models.

Various studies have demonstrated the effectiveness of bundled payment models in reducing the costs of TJA [16, 17]. The CMS has reported their findings from the BPCI Advanced Model for the first two years and found surgical savings of 3.6% compared to cost without the bundled model, driven mainly by orthopedic procedures [13]. Moreover, CMS reported improvements in the quality of care given reduced unplanned readmissions and reduced post-acute care utilization [13]. In examining approaches to successfully manage bundled payments, Froemke et al. reported savings of over $250,000 with an estimated 62% of patients coming in or under the target price [16]. These savings were a result of shorter lengths of hospital stay, more home discharges, and lower postoperative resource utilization [16]. To achieve these results, the authors standardized their care pathway to ensure the same supplies and techniques were consistently utilized [16]. Financial incentives for physicians meeting targets on quality, operation time, length of stay, and patient participation in preoperative education classes may have further led to the recorded successes of this pilot program [16]. Another study by Whitcomb et al. created a pilot program for THA bundled payments including a clinical model that based the number of physical therapy visits allocated on the discharge date [17]. For example, those discharged on postoperative day two were given 8 home physical therapy visits, those discharged three days after surgery were given 6 visits, and on the day of surgery discharge were given 4 visits included in the bundled payment [17]. The 45 THA patients included in the payment bundle clinical model were more likely to be discharged home, had a shorter LOS, and decreased overall surgical costs including post-acute care and post-hospital cost when compared to those in the pre-pilot period [17]. Both studies have noted decreased costs due to greater home discharge as opposed to SNF utilization [16, 17]. Our study was built upon these results, suggesting that opportunities to standardize postoperative therapy protocols exist, which may lead to cost savings without compromising postoperative outcomes.

Physical therapy is often an essential component of the preoperative and postoperative care of THA patients. However, predicting which patients require formal PT and utilization patterns is difficult given the multitude of factors influencing recovery. Zeng et al. developed a predictive model to determine the physical therapy placement for total knee arthroplasty, home health service or outpatient PT [28]. This model found four predictors including increased age, female gender, lack of access to transportation, and lack of motivation to participate in outpatient PT as predictive of home health care rather than outpatient therapy utilization [28]. Further, Klement et al. found increased Charlson Comorbidity Index (CCI) scores, increased body mass index (BMI), and increased preoperative Short Form 12 (SF-12) mental scores to be independent predictors for outpatient, rather than in-home PT [8]. In addition to the risk factors described by prior studies, we identified female, non-white, sleep apnea patients to be increased utilizers of postoperative therapy. While we observed no improvement in outcomes in these high utilizers, it is also important to note that this population did not experience outcomes inferior to those utilizing fewer than 16 therapy sessions. It is therefore possible that increased therapy did have a protective effect in this population, enabling them to achieve early functional improvements and low complication rates similar to lower-risk patients. In the context of potential bundled payment models, these factors should be considered for the risk adjustment of payments. It is critical that sex, race, and comorbidities be included in risk adjustment methodologies to adequately compensate providers and avoid the adverse effects of cherry-picking only patients likely to utilize few sessions. We see cost savings opportunities if therapy centers are aligned in a value-based bundle.

Recent studies have evaluated new models of physical therapy, including home therapy or remote therapy. One study by Menon et al. investigated a pilot program Outpatient Physical Therapy Home Visits (OPTHV) to improve postoperative outcomes and resource utilization [9]. The OPTHV program offers additional in-home services including preoperative physical and environmental assessments for fall risks and equipment usage, home exercises, and stair and transfer training [9]. Postoperatively, this program includes in-home PT assessments within a week after surgery and helps initiate the transition to outpatient PT while also evaluating prevention measures to avoid complications and readmissions [9]. Menon et al. found those enrolled in the Outpatient Physical Therapy Home Visits program had shorter LOS and were more likely to be discharged home [9]. Mahomed et al. randomly assigned 234 TJA patients to either home-based or inpatient rehabilitation and found no significant differences between groups in pain, functional outcomes, or patient satisfaction [29]. Another study conducted a randomized control trial of 120 THA patients for a self-directed home exercise program for 10 weeks or standard protocol that included 2 weeks of in-home physical therapy visits followed by 8 weeks of formal outpatient physical therapy [10]. This study found no statistically significant differences in functional outcome measures between the two groups [10]. Although some authors have proposed no formal outpatient physical therapy following THA, most surgeons use therapy for their patients [7, 10, 11, 30]. Further, self-directed home exercise programs are not suitable for all patients. Therefore, payment bundles should be considered for outpatient physical therapy programs to help mitigate costs without jeopardizing the quality of care.

Our study does not come without limitations. One limitation is within our outcome measures for readmission rates and emergency department visits. These measures were only recorded for institutions participating in Epic Care Everywhere, therefore patients presenting to non-Epic hospitals were not included. Second, it is possible that selection bias existed, as we only included patients who completed PT at our hospital-affiliated therapy practice, in order to collect the most accurate data regarding the number of visits, evaluations, and re-evaluations. Third, it is possible that unmeasured confounding factors, including surgical approach, hospital length of stay, and variability in adherence to PT protocols, impacted our results. Fourth, when comparing our results to those of previously published studies, the therapy protocols used across institutions were variable, and might therefore influence the different results observed. A final limitation of our study is the lack of published literature on existing outpatient PT bundles, as this payment model has not been previously implemented, to our knowledge. Further research is warranted to develop the most cost-effective PT bundled payment model, and additional investigation into adequate risk adjustment methodologies is needed.

## Conclusion

In this study, THA patients exhibited significant variability in postoperative outpatient PT utilization. While high levels of PT utilization were associated with increased costs, they did not translate to significantly improved physical function or decreased ED returns, readmissions, or returns to the OR during the 90-day postoperative period. With 75% of patients requiring 16 or fewer PT sessions, we suggest this threshold may be considered as a target for the development of future PT bundled payment models. Given the waning participation in the full episode of care bundles, the development of PT bundles may be an opportunity to increase the adoption of value-based payment models in the THA population.

## Supplementary Information

**Additional file 1. **Comorbidity definitions based on ICD-10 diagnosis codes.

## Data Availability

The datasets used and/or analyzed during the current study are available from the corresponding author upon reasonable request.

## Abbreviations

THA: Total hip arthroplasty
BMI: Body mass index
LEFS: Lower extremity functional score
ED: Emergency department
OR: Operating room/odds ratio
PT: Physical therapy
TJA: Total joint arthroplasty
TKA: Total knee arthroplasty
LOS: Length of stay
CJR: Comprehensive Care for Joint Replacement
BPCI: Bundled Payments for Care Improvement
CMS: Centers for Medicare & Medicaid Services
SNF: Skilled nursing facility
COPD: Chronic obstructive pulmonary disease
CHF: Congestive heart failure
NSAID: Non-steroidal anti-inflammatory drugs
ICD-10: International Classification of Disease 10th Edition
ASA: American Society of Anesthesiologist
HCC: Hierarchical Condition Category
OA: Osteoarthritis
AVN: Avascular necrosis
MPPR: Multiple Procedure Payment Reduction
CPT: Current procedural terminology
HTN: Hypertension
GERD: Gastroesophageal reflux disease
CCI: Charlson Comorbidity Index
SF-12: Short form 12
OPTHV: Outpatient physical therapy home visits
