# Supplementary material for: Outpatient physical therapy bundled payment models are feasible for total hip arthroplasty patients: an evaluation of utilization, cost and outcomes
Source: Arthroplasty. 2023 May 12;5:26. doi: 10.1186/s42836-023-00179-2 (PMC10176925; doi:10.1186/s42836-023-00179-2)
Supplement: Supplementary file 1 — Additional file 1. Comorbidity definitions based on ICD-10 diagnosis codes. [file 42836_2023_179_MOESM1_ESM.docx]

**Additional file 1.** Comorbidity definitions based on ICD-10 diagnosis codes

| **Comorbidity** | **ICD-10 Code(s)** |
| --- | --- |
| Type 1 diabetes | E10* |
| Type 2 diabetes | E11* |
| Type 1 or 2 diabetes | E10*–E11* |
| Sleep apnea | G47.3 |
| COPD | J44* |
| Liver disease | K70*–K77* |
| Asthma | J45* |
| AFIB | I48* |
| CHF | I50* |
| CAD | I25* |
| ESRD or CKD | N18*, I12*, I13* |
| GERD | K21* |
| Anxiety or depression | F41*, F32*, F33* |
| Primary HTN | I10* |
| PVD | I73* |
| Neoplasm | C00*–D49* |
| Nutritional anemia | D50*–D53*, D55*–D59* |

*Indicates all codes below that grouping level; COPD—Chronic obstructive pulmonary disease; AFIB—Atrial fibrillation; CHF—Congestive heart failure; CAD—Coronary artery disease; ESRD CKD—End-stage renal disease/chronic kidney disease; GERD—Gastroesophageal reflux disease; HTN—Hypertension; PVD—Peripheral vascular disease.
